# Acetylated Triterpene Glycosides and Their Biological Activity from Holothuroidea Reported in the Past Six Decades

**DOI:** 10.3390/md14080147

**Published:** 2016-08-04

**Authors:** Yadollah Bahrami, Christopher M. M. Franco

**Affiliations:** 1 Medical Biotechnology, Flinders Medical Science and Technology, School of Medicine, Flinders University, Adelaide SA 5042, Australia; 2 Centre for Marine Bioproducts Development, Flinders University, Adelaide SA 5042, Australia; 3 Medical Biology Research Center, Kermanshah University of Medical Sciences, Kermanshah 6714415185, Iran

**Keywords:** acetylated triterpene glycosides, saponins, sea cucumbers, Holothuroidea, anticancer, antifungal, immunomodulatory, holothurin, holothurians

## Abstract

Sea cucumbers have been valued for many centuries as a tonic and functional food, dietary delicacies and important ingredients of traditional medicine in many Asian countries. An assortment of bioactive compounds has been described in sea cucumbers. The most important and abundant secondary metabolites from sea cucumbers are triterpene glycosides (saponins). Due to the wide range of their potential biological activities, these natural compounds have gained attention and this has led to their emergence as high value compounds with extended application in nutraceutical, cosmeceutical, medicinal and pharmaceutical products. They are characterized by bearing a wide spectrum of structures, such as sulfated, non-sulfated and acetylated glycosides. Over 700 triterpene glycosides have been reported from the Holothuroidea in which more than 145 are decorated with an acetoxy group having 38 different aglycones. The majority of sea cucumber triterpene glycosides are of the holostane type containing a C18 (20) lactone group and either Δ^7(8)^ or Δ^9(11)^ double bond in their genins. The acetoxy group is mainly connected to the C-16, C-22, C-23 and/or C-25 of their aglycone. Apparently, the presence of an acetoxy group, particularly at C-16 of the aglycone, plays a significant role in the bioactivity; including induction of caspase, apoptosis, cytotoxicity, anticancer, antifungal and antibacterial activities of these compounds. This manuscript highlights the structure of acetylated saponins, their biological activity, and their structure-activity relationships.

## 1. Introduction

Holothurians belong to the Animal kingdom, the phylum Echinodermata, and the class Holothuroidea (from the Greek holothurion, “sea polyps”). Holothuroidea are divided into three subclasses: Dendrochirotidae, Aspidochirotacea and Apodacea. The most studied holothurians belong to the families Holothuriidae, Cucumariidae, and Stichopodidae. Holothurians are sedentary marine invertebrates, commonly known as sea cucumbers, trepang, bêche-de-mer, khiar daryaei or gamat, which vary in size from an inch in length to up to three feet long. Sea cucumbers are commercially important and have been used in Asian traditional medicine since ancient times as tonics, and frequently reported as a treatment for certain diseases; and now are gaining popularity as a dietary supplement in western countries [1]. There is a dried sea cucumber market on the Internet (www.coastsidebio.com) and over-the-counter in encapsulated form as dietary health supplements and nutraceuticals for humans and companion animals in the United States and Canada [2]. In the Qin dynasty in China, sea cucumbers were considered as a good remedy equal to ginseng for “yin deficiency of kidney, ischemia, dysentery and ulcers” [3]. Many Asians consume sea cucumbers to cure disorders including asthma, hypertension, cancer and arthritis, as well as intestinal and urinary dysfunctions [4]. Further, they believe consumption of sea cucumbers enhances the immune system and possesses aphrodisiac properties. Thus, food supplements comprising sea cucumber extracts are currently utilized in the treatment of cancer patients in Korea, Japan, China and other countries [5].

Triterpene glycosides are well-known for their cytotoxic, antimicrobial, anticoagulant, hemolytic, antiviral, antiparasitic and antitumor properties. Some glycosides can prevent the growth [6], survival, invasion [7,8], and metastasis [9] of cancerous cells; others possess immunomodulatory activity [10], or inhibit the sodium–potassium ATPase [11], and even elicit apoptosis [12,13,14]. The wide spectrum of biological properties of the triterpene glycosides, particularly in high concentrations, is preferably associated with the interaction with Δ^5(6)^ sterols of the cellular membrane resulting in a saponification that lyses the cell. The defense mechanism against predators could be due to membranotropic action of these compounds.

This study is the first comprehensive review on acetylated triterpene glycosides isolated from sea cucumbers, and describes the structure of all acetylated triterpene glycosides (145) reported to-date, and covers the effect of the acetoxy group upon the activity of glycosides and their structure-activity relationships. Therefore, the main aim of this review is to combine and summarize the literature about the structure, biological activities and distribution of acetylated glycosides from sea cucumbers.

## 2. Sea Cucumbers Triterpene Glycosides as Chemotaxonomic Markers

Sea cucumbers are a prolific source of bioactive secondary metabolites with the potential to cure or prevent several diseases. The bioactive compounds from sea cucumbers are well-known. This high chemical diversity is a potential source of nutraceutical, pharmaceutical and cosmetic agents, many of which have been of interest in pharmaceutical development. Echinoderms belonging to the class Holothuroidea generate a complex assortment of triterpene glycosides (holothurins), which are in charge of general toxicity and defense due to their membranotropic function.

The current knowledge of holothurian diversity is virtually unknown. However, over 1500 species have been reported. Biodiversity may provide chemical diversity which increases the chance of exploring novel therapeutic compounds. Chemical fingerprinting of triterpene glycosides in holothurians can give insight on the correct taxonomic position of a species. These congeners have the potential to be used as chemotaxonomic markers.

Many studies revealed that holothurians belonging to all the extant orders of the class Holothuroidea produce triterpene glycosides. Several triterpene glycosides are specific to different taxonomic groups of holothurians. These structural characteristics and features of triterpene glycosides have been applied to resolve taxonomic problems in the class Holothuroidea [15]. Sea cucumber triterpene glycosides have been used to reclassify and improve sea cucumber taxonomy as they are useful taxonomic markers [15,16]. Therefore, the occurrence of triterpene glycosides may be considered as a chemical marker and taxonomic character for this class [17]. Indeed, the combination of chemotaxonomic and morphological and conventional methods can become a strong tool to determine sea cucumber classification, phylogeny and evolution.

It has been claimed that *S. chloronotus* collected off the Great Barrier Reef produces stichoposides C, D and E and lack of their 25(26)-dehydro derivatives, while Kitagawa et al. (1981) have isolated these glycosides as well as their 25(26)-dehydro analogs from specimens of the same species collected from the Japanese coast [16]. Therefore, there is some evidence that the geographical location can have an effect on the saponin structure. However, this discrepancy might result from using different analytical instrumentation, as these compounds are major compounds that have been reported in many species of the genus *Stichopus* collected around the world.

## 3. The Structural Features of Holothurian Triterpene Glycosides

Triterpene glycosides are naturally highly polar compounds with low volatility, first discovered in higher plants. Saponins have also been reported in some marine invertebrates particularly echinoderms, octocorals and sponges. The presence of saponins in these classes is a unique characteristic among the animal kingdom, differentiating them from other echinoderms and from each other [18,19]. Saponins are complex compounds, heterosides, composed of a saccharide moiety (hydrophilic part, water-soluble), connected glycosidically to a hydrophobic aglycone (sapogenin), which has a triterpene or steroid backbone (lipo-soluble) [20,21].

These amphipathic compounds are generally perceived as highly active natural products and the sea cucumber saponins have been well characterized for their biological activities.

Indeed, the name ”saponin” originated from sapo (the Latin word for soap) since they possess surfactant properties and create stable, soap-like foams when shaken in aqueous solution [22,23]. They have been used as emulsification and foaming agents [22,24,25]. Saponins are constituents of many plant drugs and folk medicines, especially from the Orient. They are also consumed as preservatives, flavor modifiers, food additives, vaccine adjuvants and cholesterol-lowering agents.

The main characteristic feature of the holothurians is the presence of particular holostane type triterpene glycosides, and could be differentiated by several structural features. They include a number and position of double bonds in the core and lateral chain of the aglycone, number and position of sulfated groups in the sugar moieties, number, composition and sequence of saccharide residues in the saccharide chain, and the occurrence of hydroxy, epoxy, acetoxy and ketone groups in numerous positions of the aglycone.

**Figure 1:**
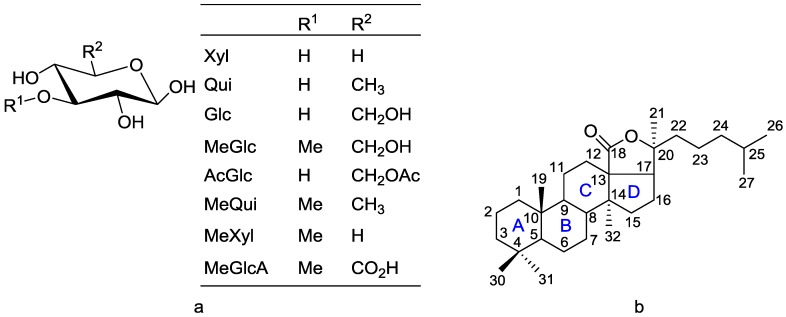
Structure of monosaccharide (**a**) and the holostane aglycone (**b**), bearing an 18(20)-lactone, the characteristic aglycone moiety in sea cucumber glycosides.

Saponins are generally divided into three main groups in accordance with their aglycone (genin) structure: triterpenoidic, steroidal and steroid alkaloid glycosides [22]. Triterpenoid saponins have aglycones that consist of 30 carbons, whereas steroidal saponins possess aglycones with 27 carbons, which are rare in nature [22]. Sea cucumber saponins, commonly referred to as holothurins, are usually triterpene glycosides containing a holostane structure, derived from lanostane, which the majority belongs to rather than nonholostane [19,21,26]. The former is comprised of a lanostane-3β-ol type aglycone containing a γ-18(20)-lactone in the D-ring of tetracyclic triterpene (3β,20*S*-dihydroxy-5α-lanostano-18,20-lactone) structural characteristic [27,28] and can contain a shortened side (aliphatic) chain, and a carbohydrate moiety consisting of up to six monosaccharide units covalently connected to C-3 of the aglycone (cyclic system) [19,29,30,31,32]. The sugar moieties mainly consist of d-xylose (Xyl), d-quinovose (Qui), 3-*O*-methyl-d-glucose (MeGlc), 3-*O*-methyl-d-xylose (MeXyl) and d-glucose (Glc), and sometimes 3-*O*-methyl-d-quinovose (MeQui), 3-*O*-methyl-d-glucuronic acid (MeGlcA) and 6-*O*-acetyl-d-glucose (AcGlc) [32]. The monosaccharide unit which is linked to the C-3 of aglycone is always Xyl, whereas MeGlc and/or MeXyl are always the terminal sugars. The monosaccharaide residues bearing 3-*O*-methyl groups are always terminal ones. The great majority of saponins have Qui or Glc as the second unit in their carbohydrate chain. Apparently, 3-*O*-methlylation of the monosaccharide units is a termination signal, preventing the further elongation of a carbohydrate moiety in sea cucumber glycosides. The basic structure of monosaccharides (a) and the holostane type aglycone (b), which is a characteristic aglycone moiety for sea cucumber saponins, are shown in Figure 1.

### Aglycones

The aglycone of sea cucumber triterpene glycosides are generally reported to contain either the 7(8) or 9(11)-double bond in their polycyclic core. Individual sea cucumber species contain one type of these aglycones in their triterpene glycosides. However, a few species of sea cucumbers have been reported to possess both types of aglycones in their triterpene glycosides. These are *Cucumaria conicospermium* [33], *Pentacta australis* [34], *Neothyonidium magnum* [35,36], *Psolus fabricii* [37,38], and *Cucumaria frondosa* [39]. The simultaneous existence of triterpene glycoside congeners having different types of unsaturation (either 7(8) or 9(11)) in the polycyclic core in some species of holothurians probably demonstrates the occurrence of common biosynthetic precursors for both type of glycosides (having 7(8)- or 9(11)-double bond). It also sheds light on the production of the polycyclic nucleus with a distinct and particular type of unsaturation at the initial stages of biosynthesis followed by oxidative transformations. Moreover, it has also paved the way that the biosynthesis of the aglycone moiety does not precede glycosylation, but is concurrent [40]. Generally aglycones with a Δ^9(11)^ double bond are characteristic of sea cucumbers belonging to the family Holothuriidae of the order Aspidochirotida, whereas those with a Δ^7(8)^ configuration are usually found in the order Dendrochirotida. Only Nobiliside A was described to contain a 7(8),9(11)-diene cyclic core [41]. However, an aglycone with such a structure (having both unsaturated bonds) is very unlikely.

The glycosides from an individual sea cucumber species may have both holostane aglycones, i.e., containing 18(20)-lactone characteristic for most of the identified sea cucumber glycosides, and nonholostane aglycones having no such lactone. They may also contain aglycones with saturated or unsaturated side chains.

The typical position of a ketone group in the sea cucumber triterpene glycosides is C-16 [15]. Thirty eight different structures have been described for the side chain of acetylated aglycones. Cucumarioside A_1_-2 is the only example of acetylated saponins with an acetyl moiety (6-OAc) linked to the terminal Glc of sugar residue [42] which is an unusual structure for a sea cucumber glycoside.

## 4. Distribution of Saponina

Over 700 triterpene glycosides have been reported in a wide range of sea cucumbers species collected from many areas including tropical Pacific, Indian, and Atlantic Oceans, the Mediterranean Sea, South America, the North Atlantic, North Pacific and Persian Gulf [43]. The existence of these compounds has been reported in all sea cucumber species studied. These glycosides are classified into four main structural categories based on their aglycone moieties: three holostane types containing a (1) 3β-hydroxyholost-9(11)-ene aglycone skeleton; (2) a 3β-hydroxyholost-7(8)-ene skeleton and (3) an aglycone moiety different to other two holostane type aglycones, and a nonholostane aglycone. The majority of saponins belong to the holostane type. Three of these possess a holostane skeleton, featuring a C-18/C-20 lactone. The presence of a 7(8) double bond alone is more characteristic of glycosides found in the family Stichopodidae. However, it is also reported in the family Holothuriidae which could represent a parallel and independent evolution or the mosaic type of biosynthesis of glycosides (independent sequence of reactions) in different taxa of sea cucumbers. It was stated that the biosynthesis of glycosides might occur through two pathways; the mosaic type of biosynthesis and the regulatory type of biosynthesis (a strictly determined sequence).

Having a nonlinear hexasaccharide chain is a characteristic feature of most of the major identified glycosides in the family Stichopodidae. Moraes et al. [16] categorized representatives of the family Stichopodidae into two groups on the basis of their glycoside compositions. The first group included species having Stichoposides and Thelenotosides and comprised *S. hermanni*, *S. variegatus,* *S. chloronotus*, *Astichopus multifidus*, *T. ananas* and *T. anax*. The second included species producing Holotoxins and contained *P. californicus* and *Apostichopus japonicus*. Nonetheless, our analysis could not confirm this observation as some common saponin congeners such as Holotoxin A_1_ were found in the viscera of *S. hermanni* [44]. Furthermore, the major triterpene glycosides (hexasaccharide) were common among the species belonging to these genera. In other words no distinction was seen between these two groups, and this species showed characteristics of both groups. Most of the identified glycosides had an oxidized group at C-23 of the lateral chain of the aglycone and an 18(20)-lactone structure, and contained non-sulfated sugar residues.

*S. hermanni* species mostly produced triterpenoic oligoglycosides lacking a sulfate group in their sugar units. In fact having a hexasaccharide chain increased the hydrophilic property of saponin congeners proposing they could be released in water surrounding sea cucumbers. *Stichopus* spp. and *Thelonota* spp. produce triterpene glycosides lacking a sulfate group in their sugar moieties.

Mass spectrometry (MS), in combination with the literature, is a rapid, sensitive, reliable and accurate technique to elucidate the structure of saponins [32,43,45]. In the positive MS analysis of saponins, isotopic distribution of the molecules can be detected around every cluster including the sodium and proton adducts, which are the most abundant species. However, the presence of acetate adducts clusters in the negative mode of ESI can be commonly observed. It is notable that this phenomenon is a typical characteristic future of using this type of ionization vehicle, and it should not be considered as acetylated compounds (ions) instantly. To ascertain whether they are acetylated compounds it is recommended to corroborate the data by conducting an MS analysis in the positive ion mode.

The notable abundance and diversity of sea cucumber saponins (more than 50 in one species) makes the purification of saponins from their natural sources a challenge. The high complexity of the saponin mixtures makes their structure elucidation and the evaluation of their potential biological activity difficult.

## 5. Acetylated Triterpene Glycosides

Decoration of saponins with acyl groups (acetoxy) contributes further structural diversity to the original saponins, which could be vital for the bioactivity of the compounds. Another structural feature that has been found only in this series of aglycones is the presence of an acetoxy group at C-16 and/or in the lateral chain of the aglycone (C-22 or C-23 and/or C-25). Based on a comprehensive literature research, acetylated saponins are mainly reported in the family Cucucmarridae; acetylated saponins are mainly identified in the orders Dendrochirotida and Aspidochirotida. *Kolga hyaline* is the only species of the order Elasipodida, reported to contain an acetylated triterpene glycoside [46]. However, the presence of acetylated saponins from the genus *Holothuria* is very rare, and only reported for *Holothuria lessoni*, *H. forskalii*, *H. nobilis*, *H. hilla*, *H. fuscocinerea*, *H. (Microthele) axiloga* and *H. pervicax*.

**Figure 2:**
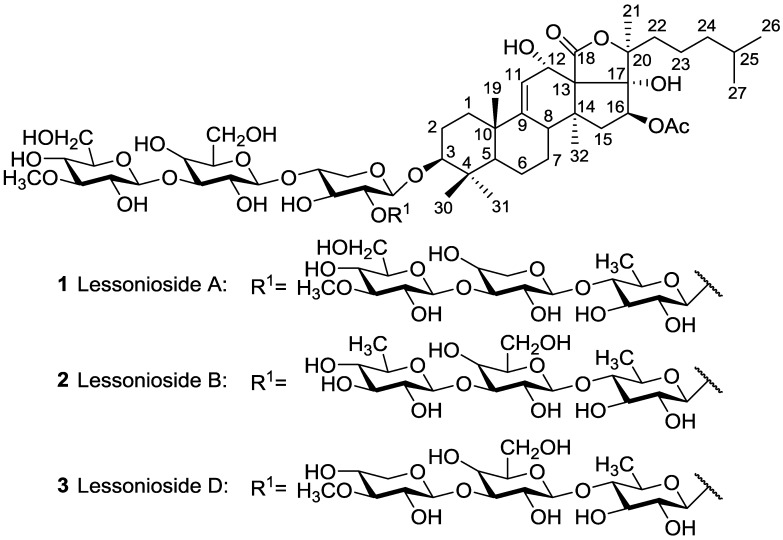

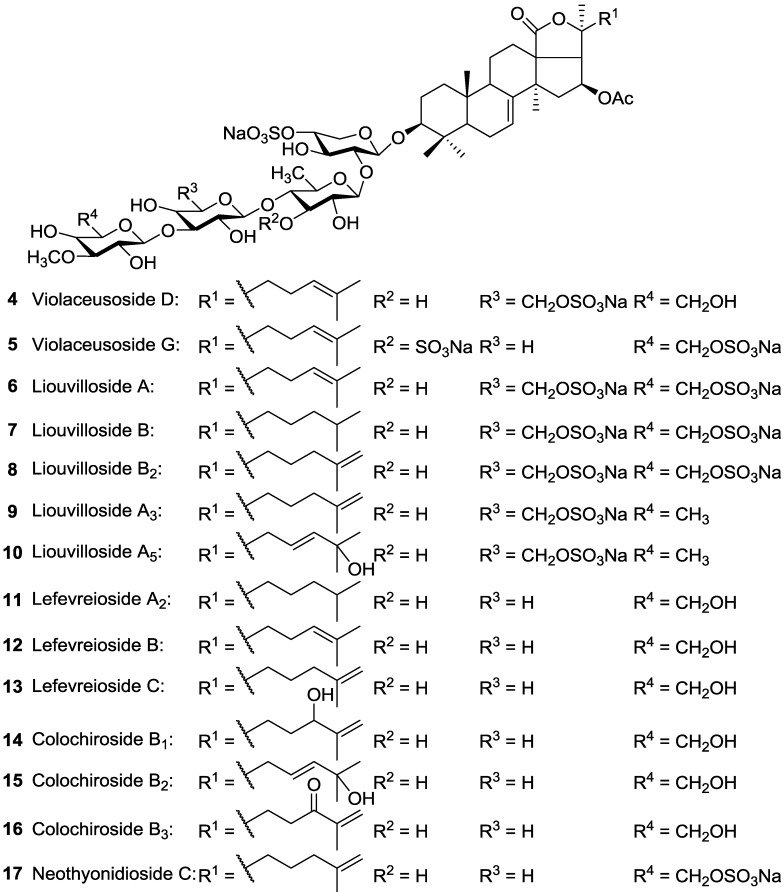

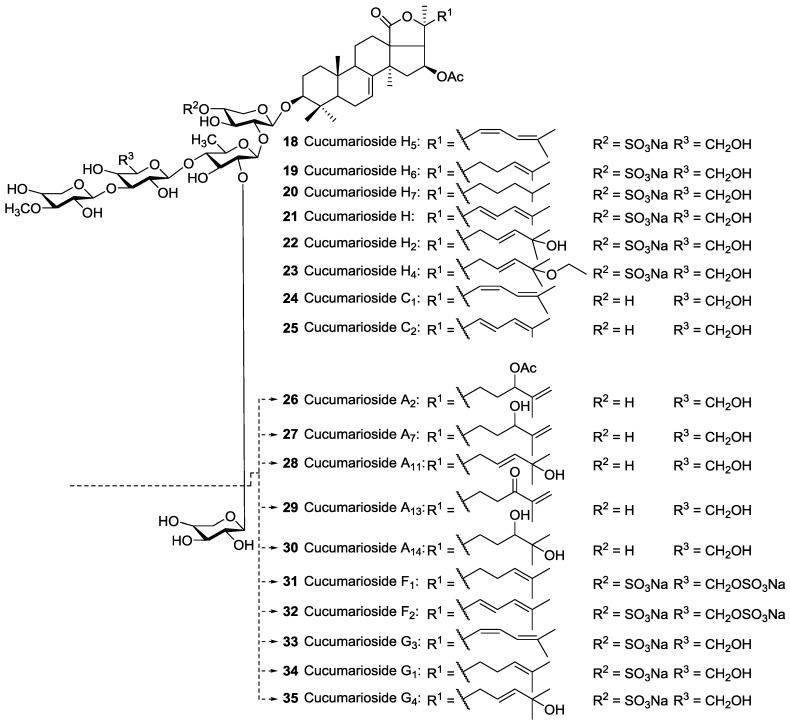

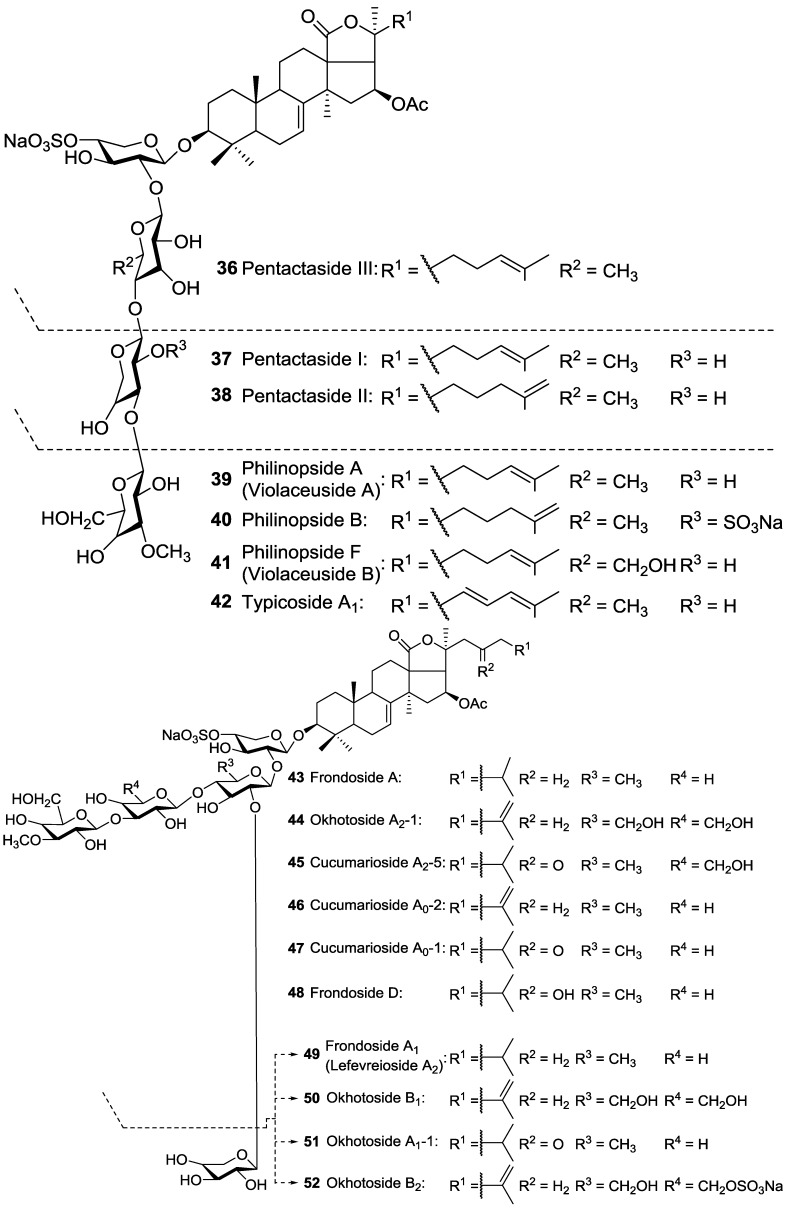

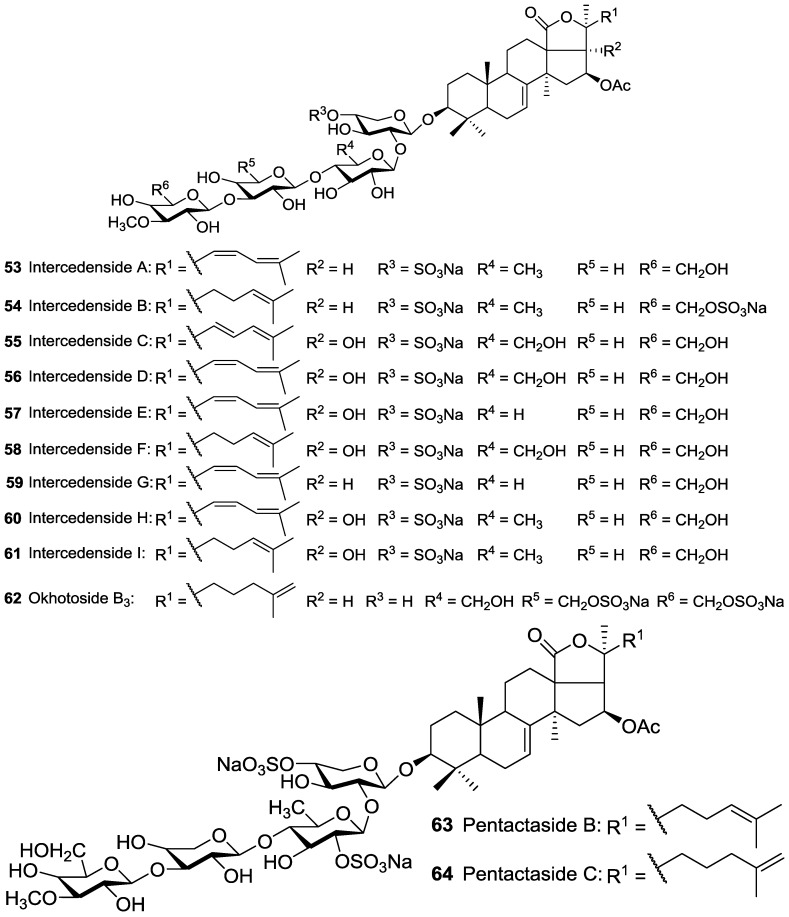

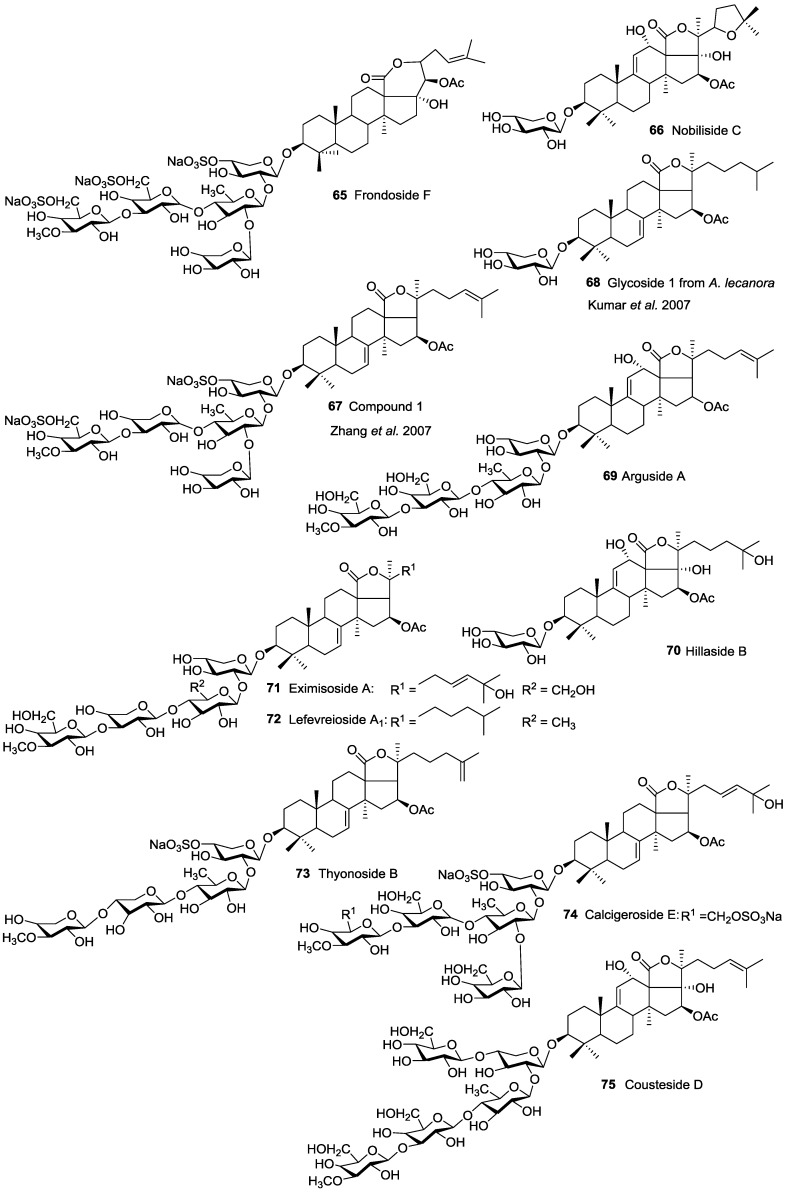

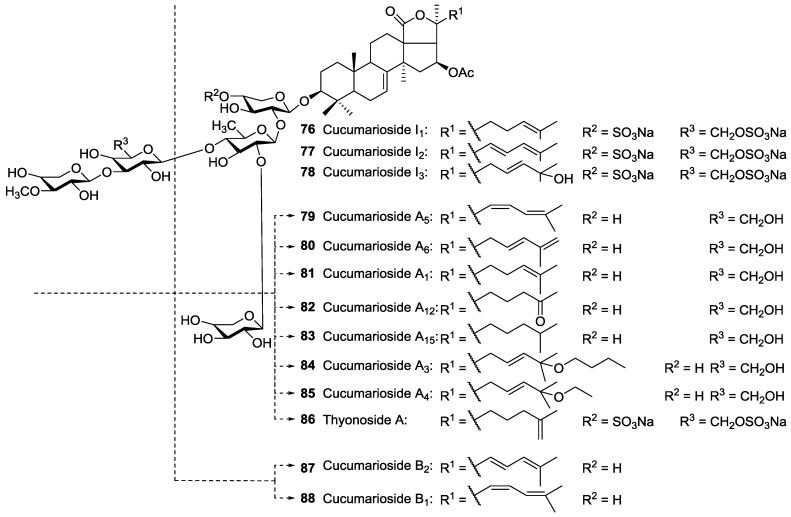
Acetylated triterpene glycosides bearing an acetoxy group at C-16 of their aglycones.

The position of acetoxy group at the aglycone creates a huge structural diversity of triterpene glycosides. Based on the position of the acetoxy group, we divided the acetylated saponins in three main groups. The first group contains an acetoxy group linked to the cyclic core of holostane type aglycone (C-16), the second group possesses an acetoxy moiety in their lateral chains of holostane type aglycone including C-22, C-23, C-24, C25, and the third group comprises of non-holostane aglycones. The majority of acetylated saponins (roughly two third) were in the first group bearing an acetoxy moiety at C-16 (Figure 2). Nobiliside C **66** is the only example of an acetylated triterpene glycoside containing a cyclic lateral moiety, while other glycosides possess a linear side chain.

**Figure 3:**
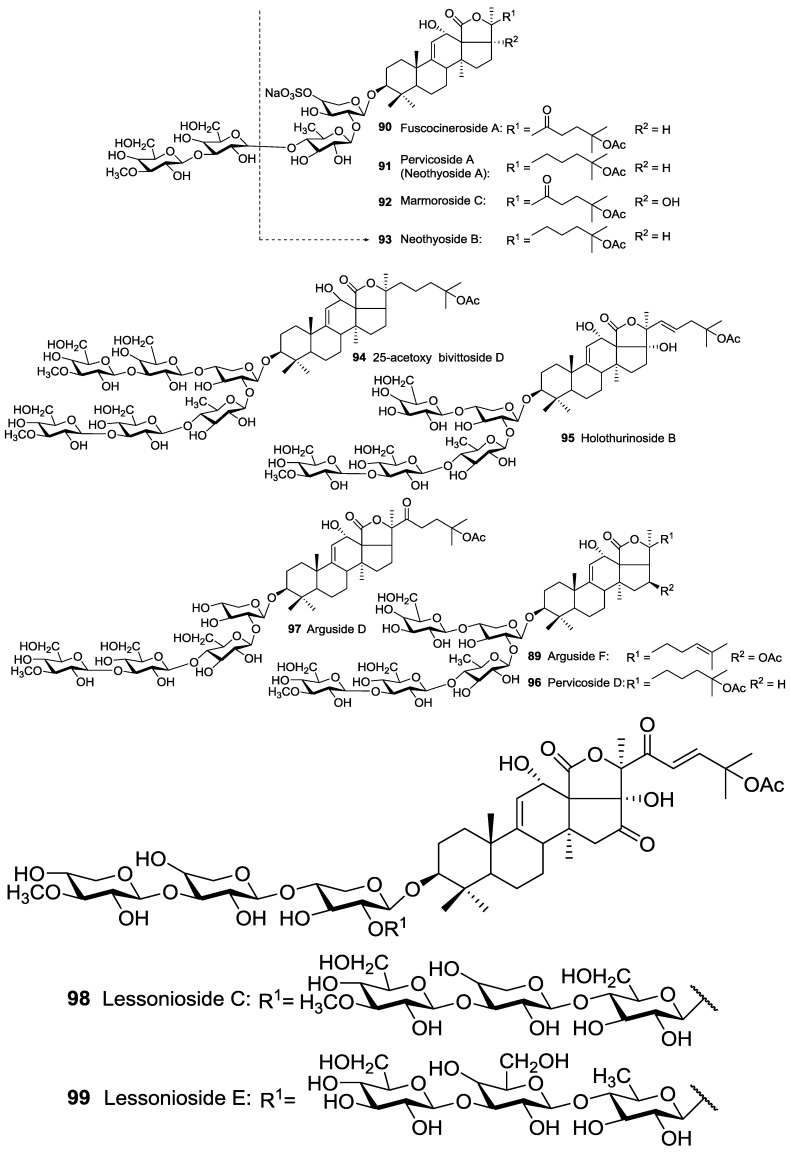
Acetylated triterpene glycosides possessed an acetoxy group at C-25 of their aglycones.

**Figure 4:**
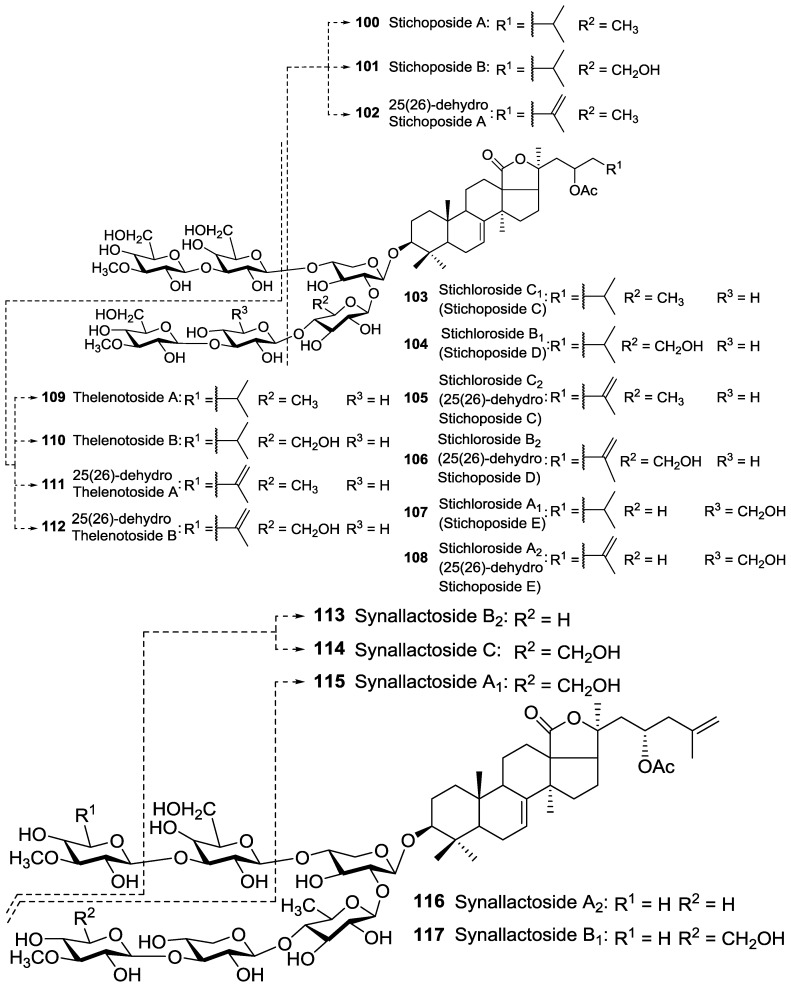
Acetylated triterpene glycosides bearing an acetoxy moiety linked to C-23 of their aglycones.

The second group contains an acetoxy group at the side chain of their aglycone (C-22, C-23, C-24 and C-25). For instance, Fuscocineroside A **90**, Pervicoside A (Neothyoside A) **91**, Marmoroside C **92**, Neothyoside B **93**, 25-acetoxy bivittoside D **94**, Holothurinoside B **95**, Pervicoside D **96** and Arguside D **97** possess an acetoxy group at C-25 of the lateral chain of aglycone (Figure 3), whereas many of other glycosides consist of an acetoxy group attached to C-23 (Figure 4).

**Figure 5:**
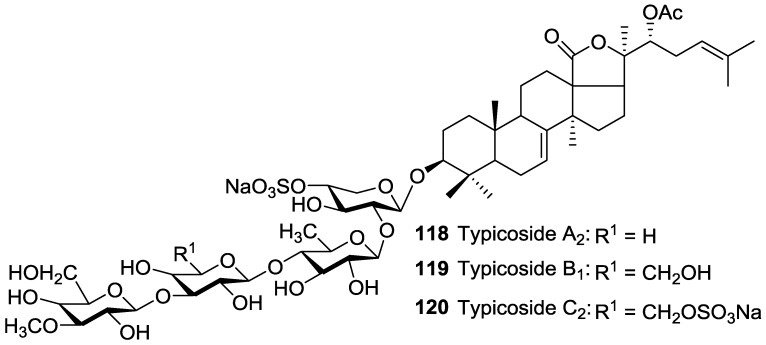
Acetylated triterpene glycosides having an acetoxy moiety linked to C-22 of their aglycones.

**Figure 6:**
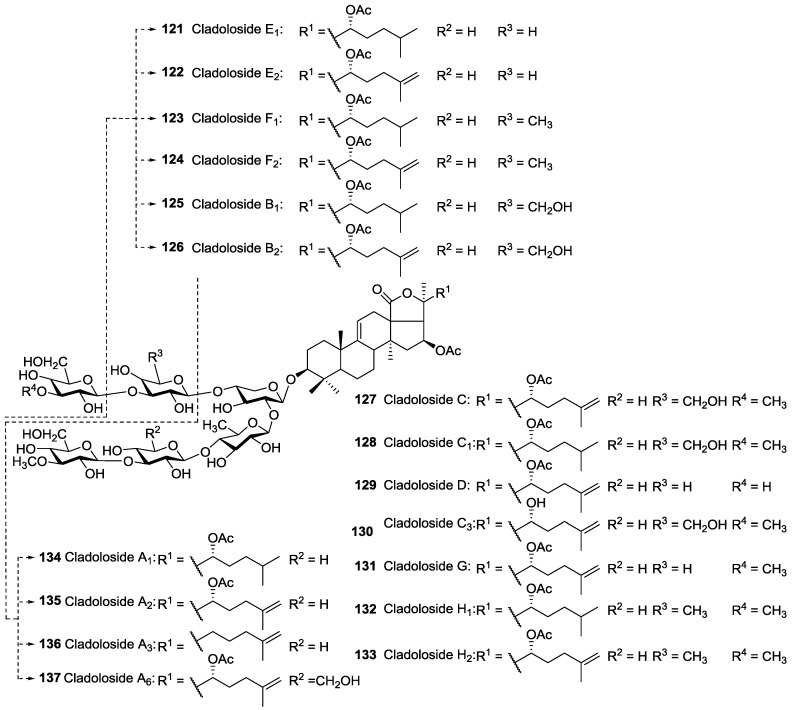
Acetylated triterpene glycosides contained two acetoxy groups at C-16 and C-22 of their aglycones.

A number of acetylated saponins have an acetoxy residue linked to C-22 of aglycone Figure 5 and Figure 6.

The majority of reported acetylated saponins possess only one acetoxy group in their structure, whereas saponins containing two *O*-acetic groups in their aglycone moieties have also been reported (Figure 6) [47].

Glycosides bearing two acetyl groups in their aglycone were reported from *C. schmeltzii* namely Cladolosides which possess 16,22-di-*O*-acetylated holostane aglycones with saturated or 25(26)-unsaturated lateral chains. In addition, Cucumarioside A_2_ **26** was also reported to have two acetoxy moieties linked to C16, and C24 of aglycone, which is the only example of acetylated saponins bearing an acetoxy group at C-24 (Figure 2).

The comprehensive list of names of the acetylated saponin congeners from sea cucumbers, the site of collection, together with their taxonomic information is summarized in Table 1. This table also indicates a few saponin congeners bearing a non-holostane aglycone.

## 6. Sulfated-Acetylated Compounds

Almost half the identified triterpenoid glycosides contain a sulfate group at the C-4 of the first xylose, and are called sulfated glycosides [123]. While the majority of sulfated glycosides contain a sulfate group at their Xyl residue, glycosides with sulfate groups bind to Glc, MeGlc, Qui and Me-Qui residues have also been reported. Some acetylated compounds also contained a sulfate group bonded to their sugar residues. For instance, Pervicoside A **91** from *Holothuria pervicax* was reported to contain a sulfate group attached to Xyl [59]. Although most of them are monosulfated glycosides, some of them are di- or trisulfated glycosides, mainly reported in the order Dendrochirotida. For instance, Liouvillosides A **6** and B **7**, from *Staurocucumis liouvillei* are described as trisulfated compounds [103]. It was contended that sea cucumbers belonging to the family Cucumariidae often contain mono-, di- and trisulfated triterpene glycosides [33]. Honey-Escandón et al. [123] stated that the ability to expel (expellability), or the absence of Cuvierian tubules and the temporal or permanent concealing habits of the species in family Holothuriidae apparently could influence the occurrence of sulfated and non-sulfated compounds in these species, which is in agreement with the Kalinin groups’ finding [67,92]. It was also stated that all the holotoxins, thelenotosides and stichoposides reported from sea cucumbers belonging to the family Stichopodidae, lack a sulfate group [16].

## 7. Non-Holostane Acetylated Saponins

The non-holostane aglycones could serve as biosynthetic precursors, the so-called “hot metabolites”, of more oxidized holostane aglycones. Sea cucumber non-holostane (lanostane) glycosides are rare and, indeed, only 24 have been reported, of which eight are acetylated. They include Kurilosides A **139** and C **140**, Frondosides A_2_-7 **138**, A_2_-8 **142** and C **145**, Psolusoside B **141** and Cucumariosides A_8_ **143** and A_9_ **144**. They have been mainly found in sea cucumber species belonging to the order Dendrochirotida. Cucumariosides A_8_ **143** and A_9_ **144** contain MeXyl as terminal monosaccharide which are regarded as intermediates of glycosides biosynthesis in sea cucumbers. Psolusoside B **141** and Kuriloside C **140** were described as tetraglycoside saponins bearing uncommon non-linear carbohydrate structures. Another structural feature for Psolusoside B **141** is the presence of an unusual Glc to Glc (1→4) linkage. The presence of this type of saccharide structure is rare for sea cucumber glycosides, and their structures need to be reconfirmed.

**Figure 7:**
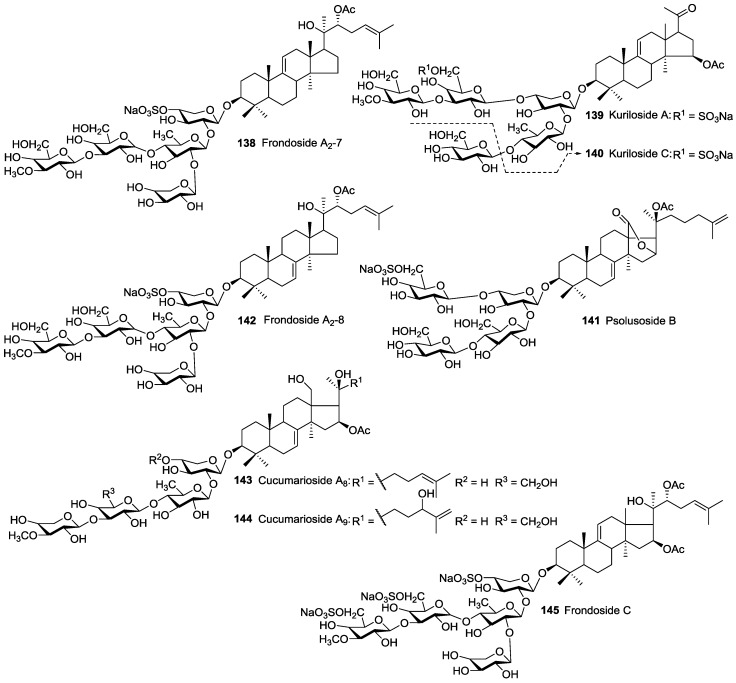
Non-holostane acetylated triterpene glycosides from Holothuroidea.

Frondosides A_2_-7 **138** and A_2_-8 **142** reported to have isomeric structure and differed from each other only in the position of a double bond in their aglycone core, with some probability they are the same compound. The structures of non-holostane type acetylated triterpene glucosides are illustrated in Figure 7.

## 8. Acetylated Saponins Having an Uncommon Structure

Our comprehensive review revealed several acetylated triterpene glycosides with unusual chemical structures. For instances, some acetylated glycosides possess trisulfated carbohydrate chains and some contain 3-MeQui as terminal monosaccharide, Figure 2.

The structures of Pentactasides I **37** and II **38** were described to contain a trisaccharide moiety which is an uncommon structural feature among holothurian glycosides and has been infrequently reported [93]. Philinopside B **40**, a disulfated tetrasaccharide glycoside, was reported to possess C-2 sulfated Xyl as third saccharide residue which is a rare structural feature for sea cucumber triterpene glycosides. Liouvillosides A_3_ **9** and A_5_ **10** were also reported from *Staurocucumis liouvillei* as disulfated acetylated tetrasaccharide glycosides bearing very rare 3-*O*-MeQui as terminal monosaccharide by Antonov et al. [102,103]. Tetrasaccharide glycoside Violaceusoside G **5** was reported to possess C-3 sulfated Qui [99], while Pentactasides B **63** and C **64** stated to contain C-2 sulfated Qui as second sugar residue in their saccharide moieties [97].

The presence of 3-*O-*MeXyl in Synallactosides A_2_ **116**, B_1_ **117** and B_2_ **113** glycosides is a rare structural feature and not characteristic for glycosides from representatives of the family Stichopodidiae.

To date, two types of glycosides having a diene-system in the side chain of their aglycones have been reported; glycosides bearing the 22*E*,24-diene system from *E. fraudatrix* and *E. pseudoquinquesemita* and glycosides having the 22*Z*,24-diene system from *E. fraudatrix* and *Mensamaria intercedens*. However, Cucumarioside A_6_ **80** was reported to contain a 23*E*,25-diene system which is an uncommon structure for sea cucumber glycosides.

A trisulfated pentaoside, Frondoside F **65**, having an 18(22)-lactone instead of 18(20)-lactone and a saturated polycyclic nucleus in the aglycone was reported by Yayli [78]. These structural characteristics are uncommon and unprecedented for sea cucumber triterpene glycosides, and the published data raise many questions. The NMR and MS findings are not supporting the proposed structure. For instance, there is no correlation between protons at C-8 and C-9 in the ^1^H–^1^H-COSY spectrum, or no peak can find for quasi-molecular ion in the FAB mass spectrum data. Bearing an acetoxy group at C-20 and having a C-18(22)-lactone are unusual structures for triterpene glycosides of sea cucumbers in addition to the absence of a double bond in the nucleus of the aglycone moiety. In addition, the lateral chain of aglycone is connected to C-22 instead C-20 [78]. Therefore the structure suggested by Yayli [78] seems to be doubtful. A possible justification for these data could be that, in terms of ‘‘frondecaside’’, the author dealt with a very complicated cocktail of glycosides contacting aglycone moieties with both 7(8)- and 9(11)-double bonds whose signals were not accumulated in the NMR spectra. Furthermore, the author pointed out Frondosides A **43** and A_1_ **49** as isomers, which is not correct [78], since they differ from each other in the numbers of monosaccharide units and are not isomers.

The structure of Thyonoside B **73** reported from *Thyone aurea* also appears to require additional confirmation [107] in order to be confirmed and accepted. In this glycoside, the terminal 3-*O*-MeXyl is linked to C-4 of the third monosaccharide unit (xylose) instead of C-3, which is described in all previously identified sea cucumber glycosides without exception.

## 9. Immunomodulatory Properties of Acetylated Saponins

It has been reported that triterpene glycosides isolated from sea cucumbers possess potent immunomodulatory activity. For instance, Frondoside A **43**, reported as the major triterpene glycoside from sea cucumber *Cucumaria frondosa,* possesses immunomodulatory properties [124]. This group suggested that Frondoside A **43** induced prolonged lysosomal activity of mouse macrophages in vivo and in vitro with different concentrations, and stimulated the phagocytosis process of macrophages. Aminin et al. [124] demonstrated that Frondoside A **43** stimulated cell-based immunity namely phagocytosis with a very weak effect on the humoral immune response. In contrast to Frondoside A **43**, Cumaside (a non-toxic complex of monosaulfated glycosides mainly Cucumarioside A_2_-2 from *Cucumaria japonica*) was stated to have a remarkable stimulatory effect on the humoral immune system [125]. Therefore, saponins can boost the immune system via their adjuvant properties. Monosulfated triterpene glycosides isolated from *Cucumaria japonicus* were described as the most effective immunostimulants, while di- and trisulfated saponins were reported as immunosuppressors [125].

Six monosulfated triterpene glycosides namely Frondoside A_1_ **49**, Okhotoside B_1_ **50**, Okhotoside A_1_-1 **51**, Frondoside A **43**, Okhotoside A_2_-1 **44**, and Cucumarioside A_2_-5 **45**, isolated from *C. okhotensis* were reported to increase lysosomal activity of mouse macrophages and reactive oxygen species (ROS) formation in the macrophages in which the highest stimulatory activity of the lysosomal macrophage was induced by Frondoside A_1_ **49**, Frondoside A **43**, and Cucumarioside A_2_-5 **45** [85]. In addition, Silchenko et al. [117] investigated the immunomodulatory activity of the triterpene glycosides Cucumariosides I_2_ **77**, H **21**, A_5_ **79**, A_6_ **80**, B_2_ **87**, and B_1_ **88** isolated from *Eupentacta fraudatrix* on mouse peritoneal macrophages and found only Cucumariosides I_2_ **77**, A_5_ **79**, and B_2_ **87** showing an increase in the lysosomal activity of macrophages. They reported that there is no direct correlation between lysosomal activity and cytotoxicity of the glycosides.

It might be concluded that some acetylated saponins possess a particular and selective stimulatory effect on the cellular immune system. Therefore, triterpene glycosides from sea cucumbers might introduce a new platform to cure and/or prevent diseases associated with the humoral and cellular-immune status, and could be useful as effective immunomodulatory agents.

## 10. Cytotoxicity and Anticancer Activity of Saponins

Saponins have been reported to be cytotoxic to mammalian cells primarily due to their membranolytic activity. However, this is dependent on concentration and other specific biological activities have been noted. There is a close relationship between the chemical structure of saponins and their biological activities. Observations from numerous studies confirm that the biological activity of saponins is influenced both by the aglycone and the carbohydrate moiety. The correlations between the structure and the cytotoxicity of triterpenoid glycosides have been described by several groups. It has been stated that having a linear carbohydrate chain is essential for the biological activity of saponins resulting in modifying the cellular membrane [92,126]. For instance, the presence of a linear tetrasaccharide fragment in carbohydrate chain increases the activity. In the case of triterpene saponins for example, acylation seems to increase their hemolytic potential [127]. The obtained results indicated that a free hydroxy at C-16 may be of importance in mediating cytotoxicity. However, the presence of a hydroxy at C-15 and acetyl group had a detrimental effect [127]. Generally, the presence of hydroxy group in the lateral chain of aglycone reduces the cytotoxicity of glycosides.

Triterpene glycosides retard tumor cell proliferation and stimulate apoptosis. The mode of actions could be selective inhibition by arrest of the cell cycle [128], specific cytotoxic activity affected by the saccharide moiety of the saponin structure [129], and non-specific cytotoxicity resulted from detergent function [130].

It has been reported that the presence of acetoxy groups usually enhances cytotoxic potency [130]. The effects of two monosulfated triterpene glycosides; Frondoside A **43** (acetylated) isolated from sea cucumber *Cucumaria frondosa* [76], and Cucumarioside A_2_-2 (non-acetylated) from *C. japonica* [131], on caspase activation and apoptosis of different human leukemia cells death-inducing capability were determined and compared [12]. Frondoside A **43** has an acetoxy group at C-16 of the aglycone and xylose at the third monosaccharide residue (as opposed to a 16-keto group and a Glc as the third sugar moiety in Cucumarioside A_2_-2). Therefore, the main structural differences between them are the functional group at C-16 and the third monosaccharide in their sugar chain. This study determined the acetyl group at C-16 as the structural feature responsible for increasing cytotoxicity of this compound, and showed that the presence of acetoxy group at C-16 could play a remarkable role in cytotoxicity and caspase activation of this compound [12]. Both compounds strongly induced apoptosis of leukemic cells, but the process was more rapid and potent in cells treated with Frondoside A **43** (acetylated), in comparison to Cucumarioside A_2_-2. They suggested that Frondoside A **43** results in caspase-independent cell death. Frondoside A **43** did not stimulate caspase activation before earlier apoptosis, whereas Cucumarioside A_2_-2 induced-apoptosis was caspase-dependent. Therefore, Frondoside A **43** may initiate apoptosis pathways in a caspase-independent manner. Jin et al. [12] stated that Frondoside A **43** is more toxic to the leukemia cells than Cucumariosides, and elicits apoptosis in HL-60 cells through the signal transduction perforin/granzyme pathway. However, Li et al. [13] reported that low concentrations of Frondoside A **43** stimulates apoptosis of pancreatic cancer cells through the mitochondrial and activation of caspase cascade. In addition, Frondoside A **43** was reported to hinder breast cancer cell invasion/migration by decreasing matrix metalloproteinase (MMP)-9 expression via blockage of nuclear translocation and transactivation of NF-κB and AP-1 [132]. Furthermore, Janakiram et al. [2] showed that Frondanol A5, (a glycolipid extract mixture of Frondoside A **43**, lipid and chondroitin sulfate) inhibited proliferation and activated caspase-2 inducing apoptosis of colon cancer cells. On the other hand, additional acetylation of the hydroxyl group at C-4" of rhamnose was reported to significantly decrease the cytotoxic activity [127]. Kalinin and coworkers stated that the evolution of saponins from sea cucumber led from non-holostane glycosides with branched carbohydrate chains to holostane compounds containing linear carbohydrate moieties, and this transition increased the activity of saponin significantly [60].

It was also reported that Frondoside A **43** is a selective stimulant of cell-based immunity, but had no marked adjuvant property. The aglycone of Cucumarioside A_2_-2 isolated from *C. japonica* contains a 25(26)-terminal double bond and a C-16-keto group compared to Frondoside A [124]. Hence, the small differences in the structure of saponins may impact on the level of immunomodulatory properties.

The anti-metastatic property of Frondoside A **43** was also investigated against human breast cancer cell lines [133]. The authors stated that Frondoside A blocked the expression of TPA-induced MMP-9 likely through the downregulation of AP-1 and NF-κB signaling pathways in which impeded the activation of the PI3K/Akt, ERK1/2 and p38 MAPK signals, which ultimately results in downregulation of MMP-9 expression.

The cytotoxicity of Arguside A **69**, bearing an acetoxy group at C-16, from *Bohadschia argus* was examined against four human tumor cell lines [49] which showed more activity towards HCT-116 cells than others. Furthermore, Intercedenside A **53**, having an acetoxy group at C-16, was also pointed out to possess notable in vivo antineoplastic property against mouse Lewis lung cancer and mouse S180 sarcoma [89].

It has been documented that the existence of an 18(20)-lactone, and 9(11) double bond in lanostane of the aglycone moiety, and having a linear tetrasaccharide residue in the sugar chain are very crucial for the membranotropicity of these compounds [92,134]. Triterpene glycosides bearing a 9(11)-double bond, the presence of an 18(20)-lactone in the aglycone, with at least one oxygen group adjacent to this functional moiety is substantial for their biological activity [126,132].

In general, the structure of the carbohydrate chain is also a characteristic which defines the biological activity of these glycosides. For instance, it has been reported that Stichoposide A **100** (a disaccharide glycoside) and Stichoposide E **107** (a hexasaccharide glycoside having a Xyl as the second sugar moiety) showed lower membranotropic activity than other stichoposides [92]. The presence of a sulfate group can also influence the biological activity of triterpene glycosides considerably [27]. The presence of a sulfate group attached to the first Xyl of a linear tetrasaccharide residue of triterpene glycosides increases their activity. However, the activity of sulfated glycosides will influence with the type, sequence and order of the other monosaccharides in their sugar resides as well as the position of sulfate group. Stichoposide C (Stichloroside C_1_) **103** is a quinovose-containing hexaosides at its second position. It has been described that Stichoposide C stimulated apoptosis of human leukemia and colorectal cancer cells via the activation of both intrinsic and extrinsic pathways [14]. Apoptosis was shown to be induced by this compound in mouse CT-26 subcutaneous tumor and HL-60 leukemia cells in a dose-dependent manner and resulted in the activation of Fas (CD95) and caspase-8, cleavage of Bid, mitochondrial damage, and activation of caspase-3. Stichoposide C **103** was also reported to reduce tumor growth of HL-60 xenograft and CT-26 subcutaneous tumors and enhance ceramide generation in vivo through activation of acid and neutral types of sphingomyelinases (SMase) in response to apoptotic stimuli. It should be noted that in all types of cancer cells, anticancer substances enhance ceramide levels to variable degrees [135]. Apparently, Stichoposide C **103** targets SMase which leads to an increase in ceramide and apoptosis.

Stichoposide D **104** is a hexaosides which can stimulate apoptosis of human leukemia cells through both extrinsic and intrinsic pathways; however, the potency of this induction is two to five times lower than the induction caused by Stichoposide C **103** [132]. Stichoposide D **104** by activating Fas/ceramide synthase 6 (CerS6)/p38 kinase in lipid rafts contributed to its anti-leukemic activity [136]. This group contended that the difference in only one sugar between Stichoposide C **103** and Stichoposide D **104** may impact on both the potency and the molecular mechanisms for their activities. Therefore, the number, length and the type and linkage variation in sugar moieties significantly influence the bioactivity of saponins.

Cytotoxicity of Pentactasides B **63** and C **64** from *Pentacta quadrangularis* were tested against five human tumor cell lines [97,137]. Two disulfated acetylated (an acetyl group at position 16β) holostane glycosides reported to possess significant activity against all tumor cell lines with IC_50_ values between 0.09 and 2.30 µM. Both saponins differ only in the side chain of the triterpene aglycone.

Almarzouqi et al. [7] demonstrated that Frondoside A **43** markedly reduced the growth of human breast cancer cell line MDA-MB-231 tumor xenografts in athymic mice and inhibited the migration and invasion of these cells in a wound healing assay. In addition, it provoked the caspases 9 and 3/7 cell death pathways followed by the activation of p53. Further, Frondoside A **43** was shown to have potent antimetastatic activity in mice bearing mammary gland-implanted tumors to the lungs [8].

Activation of apoptosis is the most commonly described mechanism of the anticancer drugs. Frondoside A **43** was stated to increase the activities of caspases-3 and -7 in LNM35 lung cancer cells [9], and elicits apoptosis by increasing activities of caspases-9, -3, and -7, boosting bax (an apoptotic promotor), and declining bcl-2 (apoptotic inhibitor) and mcl-1 (promoting cell survival by preventing cell death) [13], while Philinopside A **39**, an acetylated glycoside from *Pentacta quadrangularis* exhibited antitumor activity and stimulate apoptosis by inhibition of autophosphorylation of receptor tyrosine kinases [95]. In addition, they contended that Philinopside A **39** blocked all examined angiogenesis-related receptor tyrosine kinases (RTKs) widely, comprising fibroblast growth factor receptor-1 (FGFR1), vascular endothelial growth factor receptor (VEGFR), platelet-derived growth factor receptor-β (PDGFβ), as well as epithelial growth factor receptor (EGFR). Besides, Philinopside A **39** was reported to block proliferation and the migration of human microvascular endothelial cells (HMECs) in a doses-dependent manner [95].

It has been demonstrated that the lateral chain of the aglycone could impact on both the cytotoxicity against tumor cells and the selective cytotoxicity in neoplastic versus normal cells of these compounds [138]. The cytotoxicity and hemolytic activities of Cucumariosides H_5_ **18**, H_6_ **19**, H_7_ **20**, H_8_, H **21**, H_2_ **22**, A_1_ **81**, A_3_ **84**, A_4_ **85**, A_5_ **79**, A_6_ **80**, A_12_ **82** and A_15_ **83** isolated from *Eupentacta fraudatrix* were studied against mouse spleen lymphocytes and mouse erythrocytes [112,115,139]. Cucumariosides H_5_ **18**, H_6_ **19**, H_7_ **20** and H **21** differ from each other in their lateral chain of aglycones. These glycosides were reported to exhibit different cytotoxicity activities. For instance, Cucumarioside H_2_ having a 25-hydroxy group in the lateral chain demonstrated low activity against mouse spleen lymphocytes and Ehrlich carcinoma cells. Therefore, the structure of the side chain of the aglycone influences noticeably the cytotoxic property of these glycosides.

The cytotoxic potency of Cucumariosides might influence by their amphiphilicity. It has been demonstrated that the presence of a 25-OH group in the lateral chain of aglycone moiety of triterpene glycosides (Cucumarioside H_2_ **22**) decline their cytotoxicity considerably, while the Cucumariosides bearing 25-ethoxy group (Cucumarioside H_4_ **23**) possess potent cytotoxic activity against lymphocytes and very high hemolytic activity [139].

Saponins were reported to possess anti-glioblastoma effects. Research and clinical findings showed that glioblastoma is one of the most common malignant tumors in the neurological system [140]. Tian et al. [141] described the anti-glioblastoma activity of saponins from five different species of sea cucumbers and found several saponins exhibiting significant anti-glioblastoma properties in vivo by in situ administration (interstitial chemotherapy). For instance, the authors contented that Fuscocineroside A **90** exhibited in vitro cytotoxicity against human cancer cell lines HL60 (Human myeloid leukemia cells). Furthermore, Fuscocineroside A **90** could remarkably prevent the proliferation, stimulate the apoptosis and suppress the surviving expression in U251 cells (human brain) in a doses and time-dependent manner, but shows slight cytotoxicity to normal human astrocytes [141]. They inferred that saponins influence with cancer cells through multiple mechanisms of action, such as interfering with cell cycle progression, stimulating apoptosis, inciting stabilization of microtubules, as well as several signal transduction pathways [141].

22-oxo-25-acetoxy-echinoside B from the sea cucumber *Holothuria moebii* was stated to reduce the proliferation of four different glioma cells [142]. It was reported this compound induced apoptosis in human glioblastoma U87-MG cells and decreased the production of a number of glioma metabolic enzymes of glycolysis and glutaminolysis.

Other researchers also pointed out that Philinopside A (Violaceuside A) **39** and Philinopside F (Violaceuside B) **41** possess cytotoxic activity towards HL60 and BEL-7402 cell lines by cell division inhibitions [100].

## 11. Antifungal Activity

The frequency, intensity and diversity of fungal infections affecting humans range from the superficial, like dermatophytosis, to the deeply invasive, such as candidiasis, has increased. Coupled with the development of resistance strains to the current antibiotics used in the clinics, there is a pressing need to develop new antifungal substances with diverse chemical structures and novel modes of action.

Antifungal activity of a number of triterpene glycosides isolated from sea cucumbers has been reported. Kitagawa et al. reported antifungal activity of six triterpene glycosides; Stichlorosides A_1_ **107**, A_2_ **108**, B_1_ **104**, B_2_ **106**, C_1_ **103** and C_2_ **105** from the body wall of *Thelonota ananas* and *S. chloronotus*, with Stichloroside B_1_ having the highest yield of 15% of the mixture [63,143]. These compounds were also reported in the body wall of *S. hermanni* by Kobayashi et al. [65]. Wang et al. [144] also reported a potent antifungal activity of the acetylated saponin, Stichloroside C_1_ **103** isolated from *S. japonicus*.

In addition, Stichloroside A_1_ **107**, A_2_ **108**, B_1_ **104**, B_2_ **106**, C_1_ **103** and C*_2_* **105** were reported by other workers to be antifungal as are Bivittoside C and D from *Bohadschia argus* (janome-namako); from *Holothuria edulis* (akamishikiri); Holothurin A and Echinoside A from *Bohadschia graeffie* (kurote-namako) [65].

We also investigated antifungal activity of isobutanol-enriched saponins and HPCPC fractions from the viscera of sea cucumber *S. hermanni* against *Fusarium pseudograminearum, Pythium irregulare* and *Rhizoctonia solani* using a modified disc diffusion agar assay [44]. Lessonioside A **1**, previously reported in *H. lessoni* [43], isolated from this species shows strong antifungal activity toward the tested strain. Our limited results show that acetylated and mono-sulfated saponins usually exhibit higher antifungal activity compared to non-sulfated and non-acetylated compounds. Our results, though with a limited number of samples, indicate that saponins, bearing a double bond at the lateral chain of the aglycone, in particular Δ^25^, have a significant antifungal activity. This suggests that the Δ^25^ terminal double bond may increase the activity. This point of view correlates well with the data on the structure-activity relationship of triterpene glycosides reported by Wang et al. [3] who suggested that the 18(20) lactone moiety and Δ^25^ double bond may increase the activity.

The configuration and composition of the aglycone and glycone moieties appear to play a crucial role in the bioactivity. Wang’s group; however, contended that the position of double bond in the core of aglycone residue (Δ^7^, Δ^8^ or Δ^9(11)^) contributes little to the bioactivity [3,145], whereas Avilov et al. [146] claimed that the position of the double bond in the aglycone nucleus influence the activity. In addition, Yuan et al. [52] stated the presence of hydroxy or acetoxy groups at C-25 in the lateral chain of the aglycone reduces the antifungal activity of saponins. Further, it was contended triterpene glycosides bearing Qui as the second monosaccharide unit are the most active against fungi [137], which requires further research and evidence. Kitagawa et al. [147] stated that the presence of a linear tetrasaccharide moiety and a sulfate group are important for antifungal activity of saponins.

It has been reported that Thyonosides A **86** and B **73** isolated from the sea cucumber *Thyone aurea* showed interesting activity against murine tumor cell line, L1210, and herpes simplex virus type 1, HSV-1 [107].

Sea cucumbers could provide an impressive source of antifungal compounds due to saponins, which are worthy of further investigation. Therefore, more extensive research is required to understand the structure-activity relationship clearly. To conclude, the cytotoxic activity depends on both configuration of the aglycone nucleus and the sequence of saccharide moiety.

## 12. Molecular Effect of Saponins on Membranes

Many biological activities of holothurian saponins occur through their membranolytic function once a certain threshold concentration is reached. Triterpene glycosides have the ability to cause membrane perturbation; altering the membrane permeability, loss of barrier function, and the rupture of cell membrane. [60,132,148]. The interaction of glycosides (aglycone part) with the Δ^5(6)^-sterols of membranes (rafts), preferably with cholesterol (ergosterol in fungi), is the major factor, but not the only factor for the determination of the many biological activities of sea cucumber glycosides [92,149,150]. Glycosides bind to membrane sterols and create glycoside-sterol complexes in membranes, modifying its microviscosity, ion permeability and the activity of embedded membrane proteins. The formation of a complex is followed by association of these complexes into “two-dimensional micellar-type structures” within the membrane [150]. The hydrophilic sugar chains of the saponins, which are supposed to be centrally orientated in the micellar-like complex, result in the development of an aqueous pore and budding of the lipid a binary membrane due to the increased curvature stress. It is recognized that the holothuroid triterpene glycosides possess strong membranolytic function towards biological and model membranes containing Δ^5^-sterols due to formation of single-ion channels and larger pores (channels) which is the basis of hemolytic, and antifungal features of these substances, explaining the wide spectrum of their biological activities.

Triterpene glycosides influence the physicochemical properties of membranes such as stability, permeability and microviscosity of lipid bilayers and lipid-protein interaction and conformation of membrane proteins [151,152]; for instance, reduction or inhibition the activity of some membrane enzymes, in particular ATPases [60]. Such sterol/saponin interactions result in an efflux of some ions, substances of the nucleotide pool and peptides, disruption of ion homeostasis and osmolarity followed by lysis and cell death [92,149,153,154]. However, activities of sea cucumber glycosides in sub-cytotoxic doses are especially interesting. Low concentrations of triterpene glycosides from sea cucumbers and plants were reported to interfere with specific membrane transport proteins in cancer cells, parasites, bacteria, and fungi and alter their activities. For instance, Frondoside A **43** and CucumariosideA_2_-2 blocked the ATP-binding cassette (ABC) transporter (membrane transport P-glycoprotein; P-gp) and multidrug-resistance protein-1(MDR1) [155,156], in which they extrude or modify the lipophilic chemotherapeutical agents and drugs. Triterpene glycosides appear to act as competitive inhibitors for P-gp, multiple resistance-associated protein1 (MRP1), and Breast cancer resistance protein (BCRP) in cancer cells, or efflux pumps in bacteria (NorA) and fungi [156]. Because of their lipophilicity property, these terpenoids probably serve as substrates for P-gp and other ABC transporters.

Triterpene glycosides influence most of the normal functions of the membrane such as ion transport, and membrane permeability. Membrane transporters, which are modulated by triterpene glycosides, can be considered as potential therapeutic targets. Despite several studies that have investigated the membranotropic properties of sea cucumber glycosides during the past three decades; however, the molecular mechanisms of action of these compounds in biomembranes are not fully understood, so further investigation needs to be done. 

Also, glycosides containing a 7(8)-double bond and lacking a C-16 ketone group possess more hemolytic activity than those with a 9(11)-double bond and a C-16 ketone group. In addition, Mal’tsev et al. [157] stated that glycosides containing quinovose as the second monosaccharide unit exhibited more hemolytic activity than other triterpene glycosides.

The ability of some triterpene glycosides to interact with glucocorticoid receptors is another intriguing mode of function for these secondary metabolites. Glucocorticoids are a class of important steroidal hormones which mediate the regulation of a manifold of physiological processes. In mammals, they play the regulatory roles in development, metabolism, neurobiology and apoptosis [158]. Due to the characteristic and similarity of structure of saponin aglycones and steroids, therefore, several pharmacological properties of saponins including anti-inflammatory, immunosuppressive and neuroprotective effects [159] as well as stimulation of adipogenesis [160] or apoptosis, are associated to interact with receptors of glucocorticoid hormones [150].

Several published data on the structure-activity relationships stressed that there is no correlation between the ability to cause hemolysis with other known activities of saponins such as their cytotoxic properties and anti-fungal or their applicability as adjuvants [150]. In short, the formation of a complex between glycoside and membranes sterols followed by the creation of single ion channels and more large pores are fundamental for hemolytic properties of sea cucumber glycosides. Table 2. summarizes the mode of action of acetylated triterpene glycosides from sea cucumbers on the membrane transporters.

## 13. Chemical Nomenclature

This review revealed that ten acetylated triterpene glycoside compounds with identical chemical structures identified from different species of sea cucumbers have been given different names by different researchers. We have indicated the compounds having identifiable synonyms (the same structure, different names) in brackets in their chemical structures; Figure 2 to Figure 7 and Table 1. For instance, Pervicoside A **91** isolated from *Holothuria pervicax* (family Holothuriidae, order Aspidochirotida) [59] has an identical structure with Neothyoside A **91** reported in *Neothyone gibbosa* (family Sclerodactilidae, order Dendrochirotida) [122]. Another example, Violaceuside A **39** [100], had been described previously by another group from the sea cucumber *Pentacta quadrangularis* and called Philinopside A **39** [95]. These identifiable synonyms hampered the chemical identification and structure elucidation of compounds as well as distribution by genus.

In addition, Honey-Escandón et al. [123] reported thirteen non-acetylated triterpene glycosides having identifiable synonyms. A serious problem of homonyms (same name, different structures) was identified for the Nobiliside compounds [41,162]. The structures of two different compounds, Nobilisides B and C **66** sensu Zhang et al. [162] are identical to Nobilisides B and C **66** sensu Wu et al. [163]. Wu’s group also reported Nobilisides 1a and 2a, the latter consisting of the same structure as desulfated Holothurin A which is also highlighted by Caulier, et al. [164]. Later, Zhang et al. [165] ascribed the structure of two compounds, Nobilisides I (roman number one instead of capital i) and II, having the same formula as previously defined compounds, Holothurin A and Ananaside C, respectively [123].

Cucumariosides A_4_ **85** and H_4_ **23** were described to possess a very rare ethoxy moiety at C-25 of the lateral chain of their aglycone, and as the authors mentioned they are highly likely to be artefacts, formed during prolonged storage of the ethanolic extract or over extraction procedure [139], in addition to Cucumarioside H_2_ **22** which is also artefact generated by ethanol/water extraction. The presence of butoxy and ethoxy groups in the side chain of Cucumariosides A_3_ **84** and A_4_ **85**, respectively, is an unusual feature. These glycosides are very likely to be artificial products generated during the process of glycoside purification using EtOH for extraction and BuOH as a foam quencher during extract evaporation [115].

Wang et al. [144] reported the molecular formula for Stichloroside C_1_ **103** from *Apostichopus japonicus*; however, the proposed structure of this compound is not compatible with the elemental composition and molecular formula and it hindered the identification of this compound.

## 14. Conclusions

The marine environment is a rich source of natural compounds with promising therapeutic applications. This review has focused on acetylated triterpene glycosides and attempted to summarize the current knowledge on acetylated triterpene glycosides from different species of sea cucumbers. Sea cucumbers are a rich source of drugs, functional food and traditional folk medicine, especially in some parts of Asia. Sea cucumbers have been used as a tonic and medicinal food for more than 1000 years in Southern Asian countries; as an ancient Chinese medical text highlighted unspecified elements in sea cucumber can improve/repair the human immune system, relieve chronic fatigue syndrome and relieve stress and mental exhaustion.

These days, the interest in natural products has increased for cosmetics or medicine and agriculture application due to their properties, which can boost the action of active pesticides or chemotherapeutics or even reverse multidrug resistance, at least partially, of adapted and resistant cells. The combined applications of these substances with a cytotoxic or antimicrobial agent may converse resistance in a synergistic manner.

A large number of sea cucumber triterpene glycosides demonstrate noticeable anticancer properties at their sub-cytotoxic doses which might indicate mechanisms independent of their cytotoxicity pathways. Preliminary findings revealed that saponins provide their anticancer activities through a number of mechanisms including arresting cell cycles, induction of apoptosis, blocking of migration/metastasis and invasion of tumor cells, and interfering with angiogenesis via receptor tyrosine kinases. However, the detailed mechanisms of the anticancer properties of these secondary metabolites still remain unclear and not understand fully.

Despite extensive studies on triterpene glycosides in plants, our knowledge about the modes of action of marine triterpene glycosides on membrane transporters and the structure-activity relationship is limited. Therefore, a comprehensive study about the mechanisms of action of these secondary metabolites should be carried out to evaluate their potential as novel remedies for treatment of different diseases. Several triterpene glycosides have shown to possess anticancer activity by stimulating apoptosis of cancerous cells, while the detailed mechanisms controlling the anti-tumor property of the compounds have not been fully described. Although some preliminary data indict differential structural configuration leads to different biological activities, understanding the structural features determining the biological properties of holothurian triterpene glycosides and their structure-activity relationship is important for emerging marine drugs.

## Figures and Tables

**Table 1:** Distribution of acetylated triterpene glycosides in the sea cucumbers belonging to the class Holothuroidea.

Order	Family	Taxon	Glycosides (Holothurins) ^N^ = Non-holostane	Type of Genins H = Holostane N = Non-holostane	Place (Site) of Collection	References
Aspidochirotida	Holothuridae	*Actinopyga lecanora*	A novel triterpene glycoside **68**	H	Indian Ocean	[48]
Aspidochirotida	Holothuridae	*Bohadschia argus*	Argusides A **69** and D **97**	H	Sanya Bay, South China Sea	[49,50]
Aspidochirotida	Holothuridae	*Bohadschia cousteaui*	Cousteside D **75**	H	Gulf of Aqaba, Red Sea	[51]
Aspidochirotida	Holothuridae	*Bohadschia marmorata*	25-acetoxy bivittoside D **94**, Marmoroside C **92**, Fuscocineroside A **90**	H	Hainan Island, South China Sea	[52,53]
Aspidochirotida	Holothuridae	*Holothuria (Microthele) axiloga*	Arguside F **89**, Pervicoside D **96**	H	Hainan Island, South China Sea	[54]
Aspidochirotida	Holothuridae	*Holothuria forskalii*	Holothurinoside B **95**	H	Villagarcia de Arosa, Spain	[55]
Aspidochirotida	Holothuridae	*Holothuria fuscocinerea*	Fuscocineroside A **90**	H	South China Sea	[56]
Aspidochirotida	Holothuridae	*Holothuria hilla*	Hillaside B **70**	H	Dongshan Island, South China Sea	[57]
Aspidochirotida	Holothuridae	*Holothuria lessoni*	Lessoniosides A **1**, B **2**, C **98**, D **3**, and E **99**	H	near Lizard Island, Australia	[43,44]
Aspidochirotida	Holothuridae	*Holothuria nobilis*	Nobiliside C **66**	H	Dongshan Island, South China Sea	[41]
Aspidochirotida	Holothuriidae	*Holothuria pervicax*	Pervicoside A (Neothyoside A) **91**	H	Kushimoto, Japan	[58,59,60]
Aspidochirotida	Stichopodidae	*Astichopus multifidus*	Stichoposide B **101**, Astichoposide C **103**	H	Islas de Pinos, Cuba	[61,62]
Aspidochirotida	Stichopodidae	Genera *Stichopus*	25(26)-dihydro Stichoposide C (Stichloroside C_2_) **105**, 25(26)-dihydro Stichoposide D (Stichloroside B_2_) **106**, 25(26)-dihydro Stichoposide E (Stichloroside A_2_) **108**, 25(26)-dihydro Stichoposide A **102**	H	-	[60]
Aspidochirotida	Stichopodidae	*Stichopus chloronotus*	Stichoposides A **100**, B **101**, C (Stichloroside C_1_) **103**, D (Stichloroside B_1_) **104**, E (Stichloroside A_1_) **107**, Stichlorosides A_1_ **107**, A_2_ **108**, C_1_ **103**, C_2_ **105**, B_1_ **104** and B_2_ **106**, Stichloroside C_1_ (Stichoposide C) **103**, Stichloroside B_1_ (Stichoposide D) **104**, Stichloroside A_1_ (Stichoposide E) **107**	H	The Great Barrier Reef, Australia; Koetivi Island, Seychelles; Okinawa, Japan	[16,58,62,63,64]
Aspidochirotida	Stichopodidae	*Stichopus hermanni*	Stichoposides C **103**, D **104**, E **107** and their dehydro derivatives, Stichlorosides A_1_ **107**, A_2_ **108**, B_1_ **104**, B_2_ **106**, C_1_ **103** and C_2_ **105**	H	Okinawa, Japan	[65]
Aspidochirotida	Stichopodidae	*Stichopus mollis (Australostichopus)*	Stichoposides A **100**, B **101**, C **103**, D **104**	H	Wellington Harbor, New Zealand	[16]
Aspidochirotida	Stichopodidae	*Stichopus variegatus*	Stichoposides C **103** and D **104**, and their 25(26)-dehydro derivatives, Stichlorosides C_2_ **105**, B_2_ **106**, A_2_ **108**	H	The Great Barrier Reef, Australia	[63,66]
Aspidochirotida	Stichopodidae	*Thelenota ananas*	Stichoposide A **100** and its 25(26)-dehydro derivative **102**, Thelenotosides A **109** and B **110**, 25(26)-dihydro Thelenotoside A **111**, 25(26)-dihydro Thelenotoside B **112**, Stichoposide C **103** and its 25(26)-dehydro analog **105**, Stichoposides D **104**, E **107** and their dehydro derivatives **106**, **108**, Stichlorosides A_1_ **107**, A_2_ **108**, B_1_ **104**, B_2_ **106**, C_1_ **103** and C_2_ **105**	H	Albatross Rocks, Seychelles; Okinawa, Japan	[16,58,65,67,68,69,70,71]
Aspidochirotida	Stichopodidae	*Thelenota anax*	Stichoposides C **103**, D **104**, E **107**, Stichlorosides A_1_ **107**, B_1_ **104** and C_1_ **103**	H	Okinawa, Japan	[65]
Aspidochirotida	Synallactidae	*Synallactes nozawai*	Synallactosides A_1_ **115**, A_2_ **116**, B_1_ **117**, B_2_ **113** and C **114**	H	The southern part of the Sea of Japan	[72]
Dendrochirotida	Cucumariidae	*Actinocucumis typica*	Typicosides A_1_ **42**, A_2_ **118**, B_1_ **119**, C_2_ **120**, Intercedenside A **53**	H	Vizhinjam coast, Arabian Sea	[73]
Dendrochirotida	Cucumariidae	*Cucumaria (Aslia) lefevrei*	Lefevreiosides A_1_ **72**, A_2_ **11** and **49**, B **12**, C **13**	H	Galicia, Spain	[58,74]
Dendrochirotida	Cucumariidae	*Cucumaria frondosa*	Frondosides A **43**, A_1_ **49**, ^N^A_2_-7 **138**, ^N^A_2_-8 **142**, ^N^C **145**, D **48**, F **65**	H and N	Gulf of St. Lawrence Canada; Kolsky shore, Barents Sea, Iles de Mai, Quebec, Canada	[58,75,76,77,78,79,80]
Dendrochirotida	Cucumariidae	*Cucumaria japonica*	Cucumariosides A_0_-1 **47**, A_0_-2 **46**, A_1_-2 **146**	H	Gulf of Posiet, Sea of Japan	[42,81,82,83]
Dendrochirotida	Cucumariidae	*Cucumaria okhotensis*	Frondosides A **43** and A_1_ **49**, Okhotosides A_1_-1 **51**, A_2_-1 **44**, B_1_ **50**, B_2_ **52**, B_3_ **62**, Cucumariosides A_0_-1 **47**, A_2_-5 **45**	H	Okhotsk Sea near Paramushir Island,	[84,85,86,87]
Dendrochirotida	Cucumariidae	*Eupentacta pseudoquinquiesemita*	Cucumariosides C_2_ **25**, H **21**	H	Ushishir, Kurile Islands	[88]
Dendrochirotida	Cucumariidae	*Mensamaria intercedens*	Intercedensides A **53**, B **54**, C **55**, D **56**, E **57**, F **58**, G **59**, H **60**, I **61**	H	South Chinese Sea	[89,90,91,92]
Dendrochirotida	Cucumariidae	*Pentacta quadrangularis*	Pentactasides I **37**, II **38** and III **36**, Pentactasides B **63** and C **64**, Philinopsides A **39** and B **40**	H	Zhanjiang, near Guangdong, South China Sea	[92,93,94,95,96,97]
Dendrochirotida	Cucumariidae	*Pseudocolochirus violaceus (=Cucumaria tricolor)*	Violaceusides A (Philinopside A) **39**, B (Philinopside F) **41**, Violaceusosides D **4**, G **5**, Intercedenside B **54**, a new glycoside **67**, Liouvilloside A **6**, Lefevreioside C **13**	H	Sanya Bay, Nha Trang Gulf, Vietnam, South China Sea	[98,99,100]
Dendrochirotida	Cucumariidae	*Staurocucumis liouvillei*	Liouvillosides A **6**, B **7**, B_2_ **8**, A_3_ **9**, A_5_ **10**	H	Island of Bouvet and South Georgia (Antarctic)	[101,102,103]
Dendrochirotida	Cucumariidae	*Colochirus robustus*	Colochirosides B_1_ **14**, B_2_ **15**, B_3_ **16**, Lefevreioside C **13**, Violaceusides A **39** and B **41**	H	Nha Trang Gulf, Vietnam	[104]
Dendrochirotida	Cucumariidae	*Cucumaria conicospermium*	Cucumarioside A_2_-5 **45**	H and N	North Western shore, Sea of Japan	[33]
Dendrochirotida	Phyllophoridae	*Duasmodactyla Kurilensis*	Kurilosides A **139** and C **140**	N	Kurile Island, Sea of Okhotsh	[105]
Dendrochirotida	Phyllophoridae	*Neothyonidium magnum*	Neothyonidioside C **17**	H	Shores of south Vietnam	[35,60]
Dendrochirotida	Phyllophoridae	*Pentamera calcigera*	Calcigeroside E **74**	H	Peter-the-Great Gulf, Sea of Japan	[106]
Dendrochirotida	Phyllophoridae	*Thyone aurea*	Thyonosides A **86**, B **73**	H	Namibia	[107]
Dendrochirotida	Psolidae	*Psolus eximius*	Eximisoside A **71**	H	Okhotsk Sea near Paramushir Island, Kurile Islands	[108]
Dendrochirotida	Psolidae	*Psolus fabricii*	Psolusoside B **141**	N	Onekotan, Kurile Island	[38]
Dendrochirotida	Sclerodactilidae	*Eupentacta fraudatrix (=Cucumaria fraudatrix, =Cucumaria obunca)*	Cucumariosides A_2_ **26**, A_5_ **79**, A_6_ **80**, A_7_ **27**, ^N^A_8_ **143**, ^N^A_9_ **144**, A_11_ **28**, A_13_ **29**, A_14_ **30**, B_1_ **88**, B_2_ **87**, C_1_ **24**, C_2_ **25**, F_1_ **31**, F_2_ **32**, G_1_ **34**, G_3_ **33**, G_4_ **35**, H **21**, H_5_ **18**, H_6_ **19**, H_7_ **20**, I_1_ **76**, I_2_ **77**, I_3_ **78**	H and N	Troitsa Bay, Sea of Japan	[40,58,60,109,110,111,112,113,114,115,116,117,118]
Dendrochirotida	Sclerodactylidae	*Cladolabes schmeltzii*	Cladolosides A_1_ **134**, A_2_ **135**, A_3_ **136**, A_6_ **137**, B_1_ **125**, B_2_ **126**, C **127**, C_1_ **128**, C_3_ **130**, D **129**, E_1_ **121**, E_2_ **122**, F_1_ **123**, F_2_ **124**, G **131**, H_1_ **132**, H_2_ **133**	H	Nha Trang Gulf, Vietnam	[47,119,120]
Dendrochirotida	Sclerodactylidae	*Neothyone gibbosa*	Neothyosides A **91** and B **93**	H	Esperitu Santo Island, Mexico	[121,122]
Elasipodida	Elpidiidae	*Kolga hyalina*	Holothurinoside B **95**	H	Central Arctic Ocean, Amundsen Basin	[46]

^N^ = Non-holostane glycosides.

**Table 2:** Potential mechanisms of function of acetylated triterpene glycosides from sea cucumbers on cell membranes.

Species	Compounds	Mode of Action	References
*Psolus fabricii*	Psolusosides A and B **141**	Inhibit membrane transporter Na^+^-K^+^-ATPase	[11]
*Cucumaria okhotensis*	Frondoside А **43**	Membrane transport P-gp, MDR1	[155]
*Stichopus variegatus*	Stichoposides C **103** and D **104**	Inhibit membrane transporter Na^+^-K^+^-ATPase	[161]
*Stichopus chloronotus*	Stichoposide E **107**	Inhibit membrane transporter Na^+^-K^+^-ATPase	[161]
*Astichopus multifidus*	Astichoposide C **103**	Inhibit membrane transporter Na^+^-K^+^-ATPase	[161]
*Thelenota ananas*	Thelenotosides A **109** and B **110**	Inhibit membrane transporter Na^+^-K^+^-ATPase	[161]
*Cucumaria fraudatrix*	Cucumarioside G_1_ **34**	Inhibit membrane transporter Na^+^-K^+^-ATPase	[161]
